# Improved Solvothermal Synthesis of γ-Fe_2_O_3_ Magnetic Nanoparticles for SiO_2_ Coating

**DOI:** 10.3390/nano11081889

**Published:** 2021-07-23

**Authors:** Rashmi Mahajan, Subramanian Suriyanarayanan, Ian A. Nicholls

**Affiliations:** Linnaeus University Centre for Biomaterials Chemistry, Bioorganic and Biophysical Chemistry Laboratory, Department of Chemistry and Biomedical Sciences, Linnaeus University, SE-391 82 Kalmar, Sweden; ian.nicholls@lnu.se

**Keywords:** co-precipitation, core–shell nanoparticle, iron-oxide, magnetic nanoparticle, silica coated magnetic nanoparticle, solvothermal, surfactant

## Abstract

Monodisperse magnetic γ-Fe_2_O_3_ nanoparticles (MNPs) were prepared by a simple, improved, one-pot solvothermal synthesis using SDS and PEG 6000 as double capping reagents. This double protecting layer afforded better MNP uniformity (Z average 257 ± 11.12 nm, PDI = 0.18) and colloidal stability. Materials were characterized by DLS, SEM, TEM, XPS, and XRD. The use of these MNPs in the synthesis of core–shell structures with uniform and tunable silica coatings was demonstrated, as silica coated MNPs are important for use in a range of applications, including magnetic separation and catalysis and as platforms for templated nanogel synthesis.

## 1. Introduction

Magnetic nanoparticles (MNPs) are valuable tools for an increasing number of applications [1,2,3]. MNPs have found an important role in the biomedical sciences, e.g., cell sorting [4,5], therapeutics and drug delivery [6,7,8,9,10,11], cell transfection [12] and magnetic resonance imaging (MRI) [13,14,15] and in various technologies, e.g., separation [16,17], sensing [1,18,19,20,21], material development [22,23,24,25,26,27], including use in templated nanogel synthesis [28,29], and catalysis [30,31,32]. Iron oxide is the most commonly used material for MNP fabrication due to its ready availability, ease of use in synthesis, relatively low toxicity and chemical and mechanical stabilities [33,34]. MNPs are generally coated or functionalized to facilitate the attachment of target structures, e.g., proteins and nucleic acids, with the most commonly used coatings being gold, SiO_2_ [35] and polymers, e.g., PEG [34].

A variety of strategies have been developed for the synthesis of iron oxide MNPs. These include synthesis using vapor deposition, microemulsions, solvothermal conditions and co-precipitation [36,37]. The latter is arguably the most commonly employed due to the advantages of short reaction times and use of low temperatures. Nonetheless, synthesis using this method provides less control over MNP size and shape, which are factors that influence the ease of coating and magnetic properties [38]. Accordingly, synthesis methods enhancing the yield and uniformity of MNPs are desirable [39]. Recent efforts to improve iron-oxide MNP fabrication have also included the development of new MNP separation techniques including gradient magnetic separation and adaptions of the aforementioned strategies.

In the present study, we report the use of a simple surfactant-mediated, solvothermal approach for enhancing the monodispersity of iron-oxide MNPs in the 200 to 300 nm size range which, in turn, provides a basis for improved regularity of subsequent SiO_2_ coatings.

## 2. Materials and Methods

### 2.1. Chemicals and Materials

Iron (III) chloride hexahydrate (FeCl_3_·6H_2_O), iron (II) chloride tetrahydrate (FeCl_2_·4H_2_O), sodium dodecyl sulphate (SDS) and tetraethylorthosilicate (TEOS) were purchased from Sigma Aldrich (Steinheim, Germany). Sodium acetate, polyethylene glycol 6000 (PEG) and ammonia solution (25%) were from VWR chemicals BDH (Leuven, Belgium), Fluka Biochemica (Buchs, Switzerland) and Merck (Darstadt, Germany) respectively. Ethylene glycol, acetone and absolute ethanol (EtOH) were obtained from Carlo Erba Reagents (Barcelona, Spain). Deionized water (18.2 MΩ) acquired from a Milli-Q gradient water filtration system (Millipore, Burlington, MA, USA) was used for solution preparation, rinsing and extraction purpose.

### 2.2. Synthesis of Magnetic Iron-Oxide Nanoparticles by Co-Precipitation

Magnetic nanoparticles were synthesized by using a previously described co-precipitation strategy [40]. Briefly, FeCl_2_·4H_2_O (1.98 g, 9.5 mmol) and FeCl_3_·6H_2_O (5.46 g, 20.2 mmol) were dissolved in deionized water (50 mL) under continuous magnetic stirring (80 °C, 15 min). Aqueous ammonia solution (25%, 20 mL) was then added to the solution and stirred for a further 30 min, after which a black precipitate was obtained. Magnetic nanoparticles were separated from the precipitate using a permanent magnet (NdFeB, N35). The MNPs were then washed successively with deionized water (3 × 100 mL) until a neutral pH value was obtained and ethanol (3 × 50 mL). Drying in a desiccator (22 °C, 24 h, over-activated silica) afforded the black MNPs (3.5 g).

### 2.3. Synthesis of Magnetic Iron-Oxide Nanoparticles by Solvothermal Method

Magnetic nanoparticles were prepared by a solvothermal synthesis [41,42]. In a standard experiment, FeCl_3_·6H_2_O (4.5 g, 16.6 mmol), sodium acetate (7.8 g, 95.0 mmol), SDS (6.3 g, 21.8 mmol) and PEG (2.7 g) were dissolved into ethylene glycol (150 mL) under magnetic stirring (100 °C, 30 min). This homogeneous yellow solution was further transferred to a Teflon-lined stainless-steel autoclave (150 mL capacity). The reaction vessel was then sealed and maintained at 180 °C for 24 h. After the reaction, the container was cooled down to room temperature and the black precipitate was separated by the magnetic decantation. The subsequent product was then washed with deionized water (5 × 200 mL), ethanol (3 × 100 mL) and acetone (3 × 100 mL). Drying in a desiccator (22 °C, 24 h, over-activated silica) afforded the black MNPs (1.2 g). Furthermore, in a similar experiment, magnetic nanoparticles were prepared in the presence and absence of surfactants (SDS and PEG).

### 2.4. Optimized Preparation of Silica Coated Magnetic Iron-Oxide Nanoparticles

A modified version of the Stöber process was used for the silica coating of magnetic iron-oxide nanoparticles. Optimization of silica-coated magnetic nanoparticles (Sc-MNPs) was performed by varying the amount of magnetic iron-oxide nanoparticles, TEOS, base catalyst and various volume ratios of ethanol/deionized water [43], Table 1. Briefly, 100 mg of magnetic iron-oxide nanoparticles prepared by the solvothermal method were dispersed into the solution mixture of aqueous ethanol (*v/v*, 80%, 87.10 mL) and ammonium hydroxide (0.1 M, 1.40 mL) by ultrasonication process (1 min; 50% intensity; Sonifer, BRANSON, Dietzenbach, Germany). This was followed by the addition of TEOS (0.05 M, 11.50 mL) and allowed to react on an orbital shaker for complete modification (6 h). The total volume of the solution was estimated to be 100 mL. The sample was then separated by an external magnet, rinsed with deionized water several times until a neutral pH value was obtained, washed with ethanol (3 × 100 mL) and dried in a desiccator over activated silica (22 °C, 24 h).

### 2.5. MNP Physical Characterisation

#### 2.5.1. Scanning Electron Microscopy (SEM)

MNP morphologies (shape and size) were investigated with SEM using a LEO Ultra 55 instrument (Carl Zeiss AG, Oberkochen, Germany) equipped with a field emission electron gun. Initially, samples were placed on black carbon tape attached to alumina stubs and sputtered with a thin layer of palladium using a LEICA EM SCD 500 sputtering unit before being inserted in the SEM instrument. The vacuum level in the sample chamber was maintained at 10^−5^ mbar. A 3-kV potential was applied to the electron gun to generate the electron beam used to scan the samples.

#### 2.5.2. Dynamic Light Scattering (DLS)

Surface charge and particle size analyses of the MNPs and Sc-MNPs were performed on a Zetasizer Nano ZS (He-Ne laser; Malvern Instruments Ltd., Worcestershire, UK) furnished with a back-scattering detector (173°) at 25 °C. One milligram samples were dispersed in 20 mL of Milli-Q grade water and sonicated for 1 min using a probe sonifier (50% intensity). Samples were measured in a pre-rinsed dip cell in automatic mode with three runs.

#### 2.5.3. X-ray Diffraction (XRD)

X-ray diffraction patterns were measured with a Bruker D8 Advance diffractometer, using Cu-K_α_ radiation (λ = 1.54 Å). The measurements were performed at 0.5° incidence for 1.5 h at 300 K in the 10–85°, 2θ range.

#### 2.5.4. Transmission Electron Microscopy (TEM)

The surface morphology of particles was also observed through a transmission electron microscopy, TECNAI G2 Spirit (FEI Company, Hillsboro, OR, USA) with an acceleration voltage of 80 kV. The TEM samples were prepared by depositing a dilute suspension of particles on copper grids of 100 mesh coated with carbon. The TEM images were analyzed using ImageJ2 software (ImageJ2 online version from University of Wisconsin, Madison, WI, USA) to determine the size and size distribution of the nanoparticles.

#### 2.5.5. X-ray Photoelectron Spectroscopy (XPS)

XPS spectra were recorded using a Thermo Scientific K –alpha XPS with monochromatic Al Kα X-ray source (1486.7 eV) with a vacuum maintained at 5 × 10^−9^ torr. The XPS survey spectra were recorded at 200 eV pass energy, 1.0 eV/step. The high-resolution spectra of the individual elements were recorded by accumulating 20 scans at 50 eV pass energy and 0.1 eV/step. The sample measurements were performed at 400 μm spot. Data analysis and curve fitting were performed using origin software (origin lab corporation, Northampton, MA, USA).

## 3. Results and Discussion

### 3.1. Magnetic Nanoparticles

Initially, a comparison between MNPs synthesized by the co-precipitation and solvothermal methods was performed. MNP composition, particle sizes, size distributions and morphologies were evaluated.

#### 3.1.1. Magnetic Nanoparticle Synthesis by Co-Precipitation

The co-precipitation method is a simple and convenient approach to prepare MNPs, where aqueous salt solutions of Fe^2+^ and Fe^3+^ are treated with a base at elevated temperature. The general reaction scheme can be described as:

Fe^2+^ + 2Fe^3+^ + 8OH^−^ → Fe_3_O_4_ + 4H_2_O

**Figure 1:**
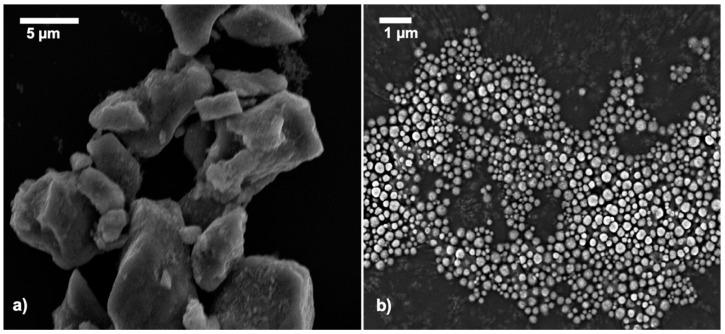
SEM images of MNPs synthesized by (**a**) co-precipitation and (**b**) solvothermal methods (spMNPs).

The main advantage of this method is that MNPs can be synthesized in good yield. However, the control of particle shape and size distribution is limited with this method [44]. A SEM image of MNPs illustrates the irregular size and amorphous nature of the particles prepared by the co-precipitation reaction (Figure 1a). This has been attributed to the lack of control over the parameters such as pH, ionic strength of the medium and reaction temperature, during the synthesis and purification steps [45,46,47]. These factors contribute to batch variability [47].

#### 3.1.2. Magnetic Nanoparticle by Solvothermal Synthesis

In this solvothermal method, FeCl_3_ is used as an iron source, which reacts with NaOAc to form the intermediate complex Fe (OAc)_3_. This is then reduced by ethylene glycol to form the iron oxide nanoparticles [48]. SDS and PEG 6000 acted as the capping layers. The adsorption and desorption of the surfactants on the crystal planes contributes the morphology of the nanoparticles [41]. A SEM study (Figure 1b), confirmed the subtle difference between the morphologies of nanoparticles prepared by coprecipitation and solvothermal. The spherical nature and monodispersity of particles observed in the latter technique. In comparison with synthesis by co-precipitation, it can be concluded that the solvothermal method as performed here using a combination of SDS and PEG 6000 provides a significantly more monodisperse magnetic nanoparticles. Further, the influence of the surfactants, alone and in concert was examined to assess the influence of this mixed protective layer on the MNP synthesis and morphology.

#### 3.1.3. Effect of Surfactants in Capping MNPs

Here, we have also explored the effect of surfactants on the colloidal stability and monodispersity of the MNPs. The influence of the surfactants on MNP morphology, charge and distribution was studied using transmission electron microscopy (TEM), scanning electron microscopy (SEM), dynamic light scattering (DLS), zeta potential (ZP) and structure properties by X-ray diffraction (XRD)

### 3.2. Material Characterization

#### 3.2.1. Surface Morphology Analysis

The size of MNPs prepared with only SDS (sMNPs) was found to be c.a. 190 nm with a narrow size distribution, as observed in the TEM (Supplementary Materials, Figure S1a). However, the surface charge of sMNPs was not reproducible as reported with zeta potential (Table 2), suggesting the surfactant layer is not consistent in capping the magnetic nanoparticles. While the reproducibility of MNPs synthesized with only PEG (pMNPs) was found to be worse than the sample prepared with the SDS (Supplementary Materials, Figure S1b; Table 2). In the case of no capping reagent, the size of MNPS (nMNPs) was about 330 nm, which is higher than other samples and agglomeration is clearly observed with TEM image, suggesting more dipole–dipole magnetic interactions among particles.

**Figure 2:**
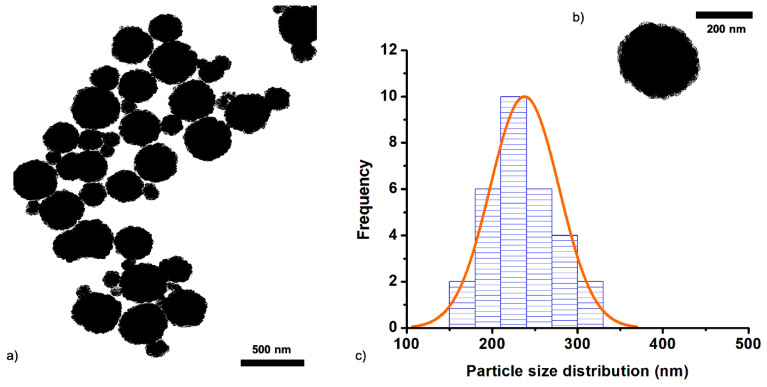
TEM micrographs (**a**,**b**) of MNPs prepared by solvothermal method using double surfactant (spMNPs) along with particle size distribution (**c**) determined with imageJ2 software.

On the other hand, MNPs prepared with the double surfactant, both SDS and PEG (spMNPs), showed the large quantity of uniform MNPs, which can be seen clearly in TEM (Figure 2) with the mean size of 230 nm, in agreement with the DLS data that revealed an average size of 257 nm with PDI of 0.18 (Table 2). The reproducibility of the average particle size and size distributions (Table 2), was found to be excellent for the spMNPs. The MNPs with double surfactants also showed colloidal stability, as no aggregates were found even after 30 d in the solvents.

Upon comparing these procedures, it can be concluded SDS or PEG alone have a slight end-capping effect on the particle size of MNPs, thus causing instability and aggregates because of magnetic dipole–dipole interactions. In the case of pMNPs, PEG interacts with the magnetic particles by hydrogen bonds only, whereas in sMNPs, the SDS interacts with particles by electrostatic interactions. On the other hand, the complex formed in the spMNPs is because of the PEG adsorption on to the surface of the micelle formed by the surfactant around the magnetic particles [41]. The dynamic force of this SDS-PEG complex is not only because of the electrostatic and hydrophobic interactions, but also the interactions between the anionic head group of the SDS and the ethylene oxide group of the polymer [49]. Therefore, this double protecting layer around the MNPs provides a steric barrier between particles, thus contributing to better colloidal stability [41].

#### 3.2.2. X-Ray Diffraction

**Figure 3:**
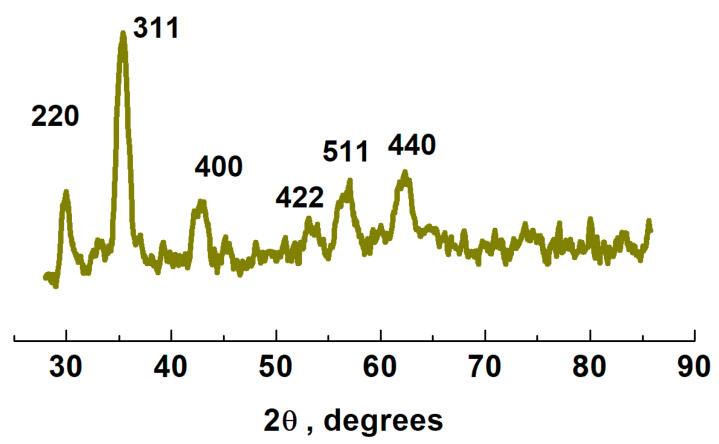
XRD pattern of γ-Fe_2_O_3_ MNPs synthesized using a combination of surfactants (SDS and PEG) at 180 °C for 24 h.

The crystalline structures of the MNPs prepared by the solvothermal method were obtained by XRD (Figure 3). From the XRD pattern of the MNP, it was found that it acquired the standard characteristics of the cubic symmetry (space group P4_1_32) of γ-Fe_2_O_3_ (PDF Card 39–1346). Maghemite structure has six characteristic diffraction peaks at 2θ = 30.12°, 35.35°, 43.08°, 53.48°, 57.92° and 63.98° which corresponds to the (220), (311), (400), (422), (511) and (440) planes of γ-Fe_2_O_3_, respectively. The crystallite size of the particles is estimated to be 24 nm (extracted from the 311 reflection) using the Scherrer’s formula. Usually, it is hard to distinguish Fe_3_O_4_ or γ-Fe_2_O_3_ only on the basis of the XRD results, because of their very similar XRD patterns. Therefore, another characterization method, X-ray photoelectron spectrum, was also used to know about the structure of iron oxides.

#### 3.2.3. X-Ray Photoelectron Spectroscopy

**Figure 4:**
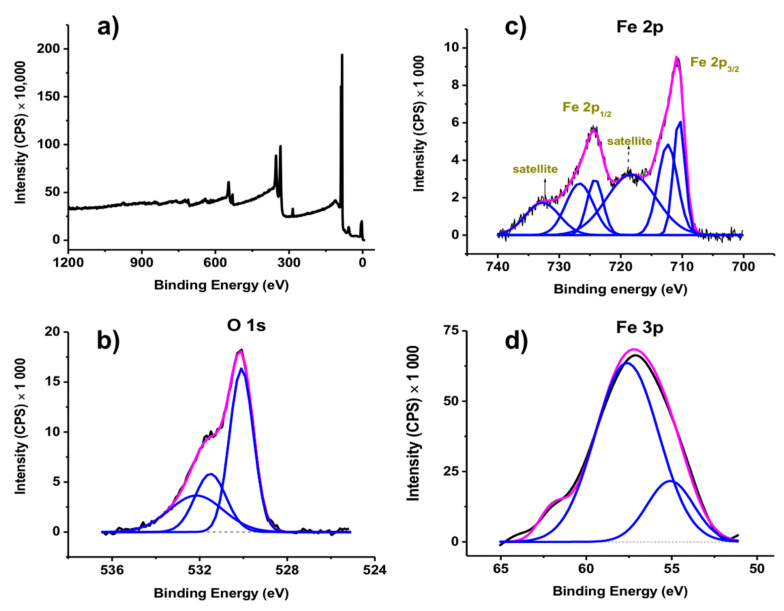
XPS spectra of γ-Fe_2_O_3_ MNPs: (**a**) survey (deconvoluted) and (**b**) O 1s, (**c**) Fe 2p and (**d**) Fe 3p core level spectra.

XPS analysis was used to determine the structure of the MNPs by examining the Fe 2p, O 1s, Fe 3p core-levels (Figure 4). The Fe 2p spectra are used to differentiate iron oxides. Here, the samples exhibit two peaks, Fe 2p_1/2_ at 724.42 and Fe 2p_3/2_ at 710.85 eV, along with their satellites at 718.44 eV and 732.78, respectively, which are characteristic of the Fe^3+^ component of γ-Fe_2_O_3_ [50,51], which helps clarify the interpretation of the XRD data presented above. This is in contrast to the binding energy of Fe 2p_3/2_ from the Fe^2+^ component in Fe_3_O_4_, which is around 708.5 eV [50,51].

### 3.3. Silica Coated Magnetic Nanoparticles (Sc-MNPs)

Silica has been considered an ideal material for the surface modification of MNPs. Silica coating has many advantages such as chemical stability and biocompatibility and can also be easily surface modified with other specific binders [34,52]. Optimization of the silica layer is important for better and uniform functional modifications. The Sc-MNPs was prepared by the Stöber technique, a seed growth mediated method for silica layer on the surface-stabilized MNPs, through the hydrolysis and polycondensation of TEOS. This reaction can take place at neutral pH, but it is accelerated under both acidic and basic conditions. The thickness of the silica layer can be modified by varying the concentration of MNPs, TEOS, catalyst and solvents (here, ethanol and deionized water). The MNPs were first dispersed in an aqueous ethanolic ammonia solution through ultrasonication. TEOS was slowly added to the MNPs dispersion, so that the MNPs could act as the seeds for deposition of the silica layer. After separation, isolation and washing of the Sc-MNPs, surface properties were SEM, DLS and zeta-potential studies were performed.

#### Surface Morphology Analysis

**Figure 5:**
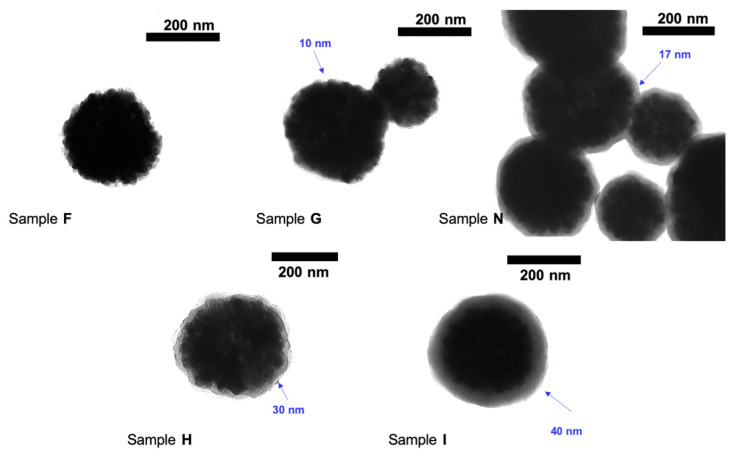
TEM images of Sc-MNPs showing an increase in the thickness of the silica layer with respect to change in concentration of aq. ammonia, sample F = 0.01 M, sample G = 0.05 M, sample N = 0.1 M, sample H = 0.25 M and sample I = 0.5 M.

Ammonium hydroxide is used as a base catalyst for fabricating the silica layer, with higher concentrations affording more regularly coated spherical MNPs. This is reflected in the average particle size increasing from ca. 245 nm to ca. 310 nm with the increase of base concentration from 0.01 to 0.5 M (Figure 5). The silica layer was negligible at 0.01 M (sample F), while the silica coating was not uniform at 0.05 M and 0.1 M (Sample G and N) of catalyst. The Sc-MNPS with the most uniform silica coating was obtained from 0.25 M ammonia (aq.) (Sample H). With even higher concentrations of the base (0.5 M, Sample I), did not lead to an increase the diameter of Sc-MNPs (the silica coating), though separate silica nanoparticles were observed.

**Figure 6:**
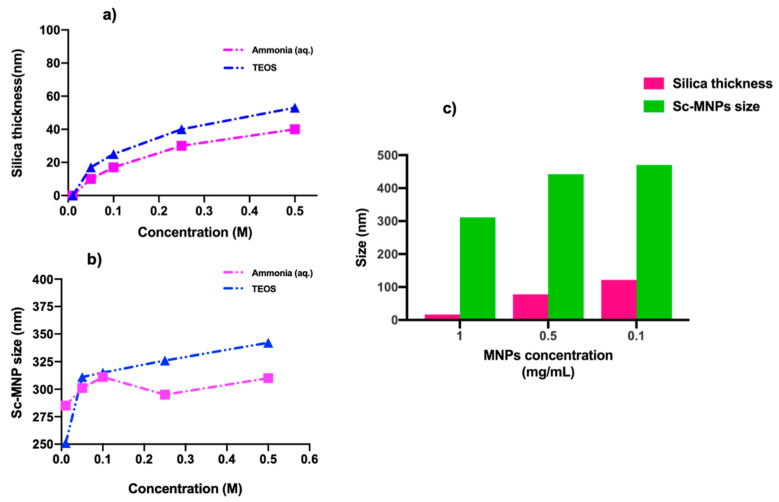
Graphs depicting the correlation between (**a**) silica thickness and (**b**) size of Sc-MNPs as a function of concentration of reagents (aq. ammonia, TEOS). (**c**) Variation of silica layer thickness (and Sc-MNPs size) with respect to the MNP concentration.

**Figure 7:**
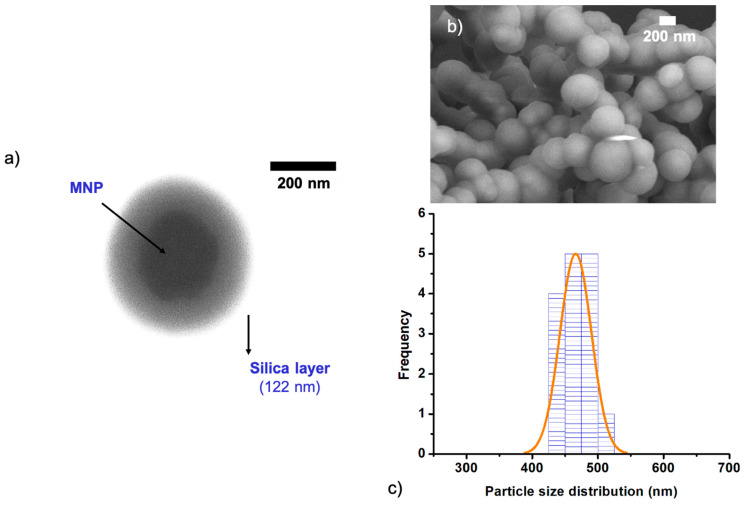
(**a**) TEM image of the sample P (0.1 mg/mL of MNPs), showing the thick silica layer around the dark iron-oxide core. (**b**) SEM image of sample P. (**c**) The particle size distribution of sample P.

The concentration of MNPs and the amount of TEOS used both significantly influence the thickness of the silica shell (Figure 6). Increasing the MNP concentration from 0.1 to 1 mg/mL, gradually decreases the shell thickness from ca. 122 to 17 nm and the particle size from ca. 470 nm to ca. 311 nm (Figure 6c). This result indicates that a uniform thick silica layer can be achieved when the concentration of TEOS is high, thus silica will homogeneously grow onto the MNP surfaces. This has been observed in two cases, sample P (0.1 mg/mL, Figure 7) and sample O (0.5 mg/mL; Supplementary Materials, Figure S3). The Sc-MNPs with a higher quantity of MNP (0.1 mg/mL, sample N), indicated that TEOS in the solution was not enough to produce a uniform silica coating on the surface of all MNPs. This trend was also observed when varying the TEOS concentration (Sample J, N, K, L, M) (Supplementary Materials, Figure S5). It was observed that the silica shell thickness ranged from effectively zero to ca. 52 nm, in the latter case accompanied by the formation of bare silica nanoparticles, when the TEOS concentration was varied from 0.01 to 0.5 M [53]. EDX confirmed the average elemental composition of the MNPs and Sc-MNPs (Supplementary Materials, Figure S4). This is the converse situation to the case above, when shell thickness increases when the MNP concentration is decreased, relative to the concentration of TEOS.

The rate of the hydrolysis reaction strongly depends on the polarity and diffusivity of TEOS in the alcohol/water mixture [43,54]. Sc-MNPs were prepared in different ethanolic solutions in water (H_2_O, EtOH, 20%, 40%, 60%, 80%; samples A, B, C, D, E, N), while keeping all other parameters constant. Both TEM and SEM images, in the case of only water (H_2_O), no silica-MNP composite, due to too fast hydrolysis and condensation of TEOS, resulting in phase separation between seed particle (MNPs) and silica [55]. Additionally, in all other samples, the concentration of TEOS (0.05 M) was too low and produced only non-uniform silica deposits on to MNPs.

Samples with TEOS concentration ≥0.10 M (K, L, M) and aq. ammonia concentration ≥0.25 M (H, I) for 0.43M magnetic nanoparticles resulted in a relatively homogeneous amorphous layer, distinctly visible on the surface of the crystalline nanoparticles, as shown in TEM studies. The increase in silica thickness was also observed in the samples with low MNPs concentration (O, P). Moreover, narrow distributions of the sizes for all this silica modified magnetic nanoparticles were found, as the standard deviations of the average length of the particles did not exceed 10%, and the particle PDIs were found to be between 0.08 to 0.3 (SI, Table 1). Studies of the magnetic properties of the MNP and corresponding Sc-MNP (sample P), revealed saturation magnetization values of M_s_ = 83.6 and 29.3 M (emu/g), respectively, which corresponds to ~35wt.% of SiO_2_ in the Sc-MNPs particles (Supplementary Materials, Figure S6). The hysteresis curves for the MNP sample were comparable to those obtained in previous studies [42,56,57,58].

## 4. Conclusions

In summary, iron-oxide nanostructures have been synthesized through two different approaches including co-precipitation and a modified solvothermal method employing a double surfactant system. The morphologies and size distributions of MNPs were characterized by TEM, SEM, DLS and ZP. Compared with synthesis by co-precipitation, the solvothermal method proved superior as it offers monodispersed spherical particles with high crystallinity (Z average 257 ± 11.12 nm, PDI = 0.18). As silica coating of MNPs is important for a range of application areas, such as sensors, drug delivery, solid phase molecularly imprinted nanogel synthesis and catalysis, the suitability of MNPs produced this way for silica coating was explored using a sol-gel approach. The results indicate that the silica layer thickness on these MNPs could be precisely controlled by varying the relative proportions of MNPs ammonia (aq.) and TEOS. Silica coated maghemite (γ-Fe_2_O_3_) core–shell nanoparticles were obtained with regular coating thickness over the range 25–125 nm.

## Figures and Tables

**Table 1:** Conditions for synthesis of Sc-MNPs.

Sample No.	MNP Concentration (mg/mL)	TEOS Concentration (M)	Catalyst Concentration (M)	Ethanolic Solution (%, *v*/*v*) EtOH in H_2_O
A	1	0.05	0.10	H_2_O
B	1	0.05	0.10	EtOH
C	1	0.05	0.10	20
D	1	0.05	0.10	40
E	1	0.05	0.10	60
F	1	0.05	0.01	80
G	1	0.05	0.05	80
H	1	0.05	0.25	80
I	1	0.05	0.50	80
J	1	0.01	0.10	80
K	1	0.10	0.10	80
L	1	0.25	0.10	80
M	1	0.50	0.10	80
N	1	0.05	0.10	80
O	0.5	0.10	0.10	80
P	0.1	0.10	0.10	80

**Table 2:** DLS (the hydrodynamic diameter (Z average); the polydispersity index (PDI)) and the zeta potential (ZP) of the MNPs prepared by solvothermal method.

Sample Name	Batch No	Z Average (nm)	PDI	ZP (mV)
nMNPs	B1	324 ± 2.99	0.192 ± 0.020	−22.60 ± 0.30
	B2	335 ± 2.26	0.200 ± 0.009	−06.70 ± 0.33
	B3	329 ± 2.27	0.207 ± 0.007	−10.70 ± 0.25
sMNPs	B1	291 ± 1.51	0.138 ± 0.005	03.52 ± 1.74
	B2	283 ± 9.01	0.166 ± 0.037	17.00 ± 1.77
	B3	307 ± 11.4	0.223 ± 0.024	07.25 ± 1.70
pMNPs	B1	173 ± 1.73	0.089 ± 0.025	14.00 ± 1.30
	B2	300 ± 4.66	0.157 ± 0.035	17.01 ± 0.85
	B3	215 ± 6.78	0.111 ± 0.019	13.03 ± 2.49
spMNPs	B1	261 ± 15.0	0.229 ± 0.027	37.70 ± 0.12
	B2	263 ± 9.30	0.166 ± 0.048	39.70 ± 3.44
	B3	247 ± 9.06	0.160 ± 0.023	41.00 ± 3.59

## Supplementary Materials

The following are available online at https://www.mdpi.com/article/10.3390/nano11081889/s1, Figure S1, TEM micrographs (500 nm scale bar) of iron oxide magnetic nanoparticles prepared by solvothermal method using (a) no surfactant (b) SDS (sMNPS) (c) PEG (pMNPS). Figure S2, SEM micrographs (200 nm scale bar) of iron oxide magnetic nanoparticles prepared by solvothermal method using (a) no surfactant (b) SDS (sMNPS) (c) PEG (pMNPS). Figure S3, TEM & SEM (inset) images of sample O (0.5 mg/mL of MNPS), with particle size distribution, showing the silica layer of 78 nm encapsulating the magnetic core. Figure S4, EDX graph confirming the presence of silica layer on the surface of magnetic nanoparticles (sample O). Figure S5, TEM micrographs of Sc-MNPS showing an increase in the thickness of the silica layer with respect to change in concentration of TEOS, sample K = 0.1 M, sample L = 0.25 M, sample M = 0.5 M. Figure S6, Magnetization hysteresis loops of MNPs (cyan) and Sc-MNP (sample P, blue) at 300 K with magnified loops inserted (H, magnetic field; M, magnetization). Table S1, Data exhibiting polydispersity index (PDI) of the silica coated magnetic nanoparticles obtained by dynamic light scattering. Table S2, Comparison of material properties with those from previously reported studies. Reference [59] is cited in the Supplementary Materials.
