# Supplementary material for: Improved Solvothermal Synthesis of γ-Fe2O3 Magnetic Nanoparticles for SiO2 Coating
Source: Nanomaterials (Basel). 2021 Jul 23;11(8):1889. doi: 10.3390/nano11081889 (PMC8398533; doi:10.3390/nano11081889)
Supplement: Supplementary file 1 [file nanomaterials-11-01889-s001.zip › nanomaterials-1294907-supplementary.pdf]

nanomaterials

MDPI

Supplementary Materials

Improved Solvothermal Synthesis of γ-Fe_2O_3 Magnetic Nanoparticles for SiO_2 Coating

Rashmi Mahajan *, Subramanian Suriyanarayanan * and Ian A. Nicholls

Linnaeus University Centre for Biomaterials Chemistry, Bioorganic and Biophysical Chemistry Laboratory, Department of Chemistry and Biomedical Sciences, Linnaeus University, SE-391 82 Kalmar, Sweden; ian.nicholls@lnu.se

* Correspondence: rashmi.mahajan@lnu.se (R.M.); esusu@lnu.se (S.S.)

Contents

1. Magnetic Nanoparticles Prepared by Solvothermal Method 2
2. Effect of Different Concentration of MNPS in Silica Coating 4
3. Effect of Various TEOS Concentration on Silica Coating 6
4. PDI of the Sc-MNPs 7
5. Magnetic Hysteresis Curves 8
6. Comparative Studies 9
References 9

1. Magnetic Nanoparticles Prepared by Solvothermal Method

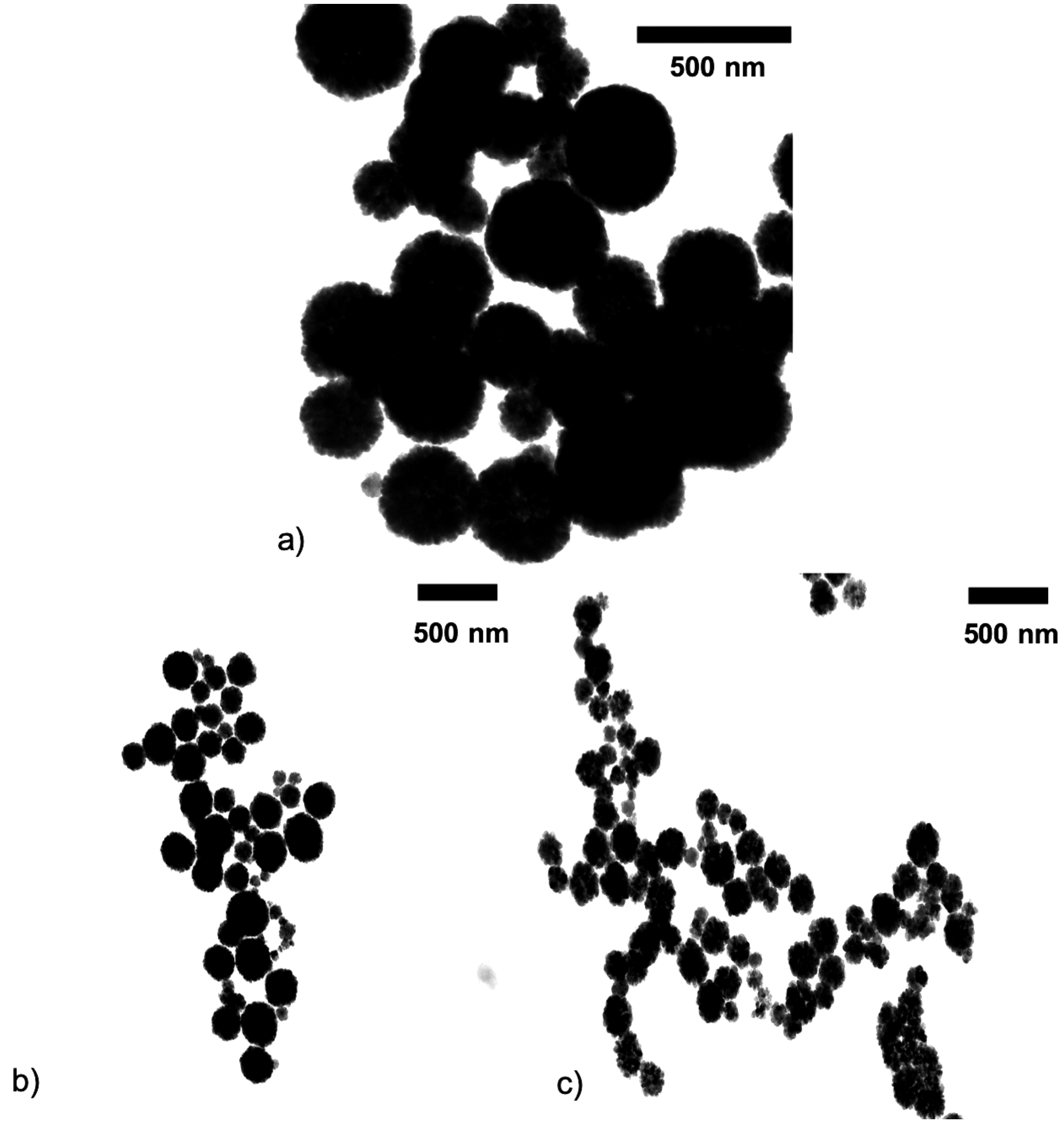

Figure S1. TEM micrographs (500 nm scale bar) of iron oxide magnetic nanoparticles prepared by solvothermal method using (**a**) no surfactant (**b**) SDS (sMNPS) (**c**) PEG (pMNPS).

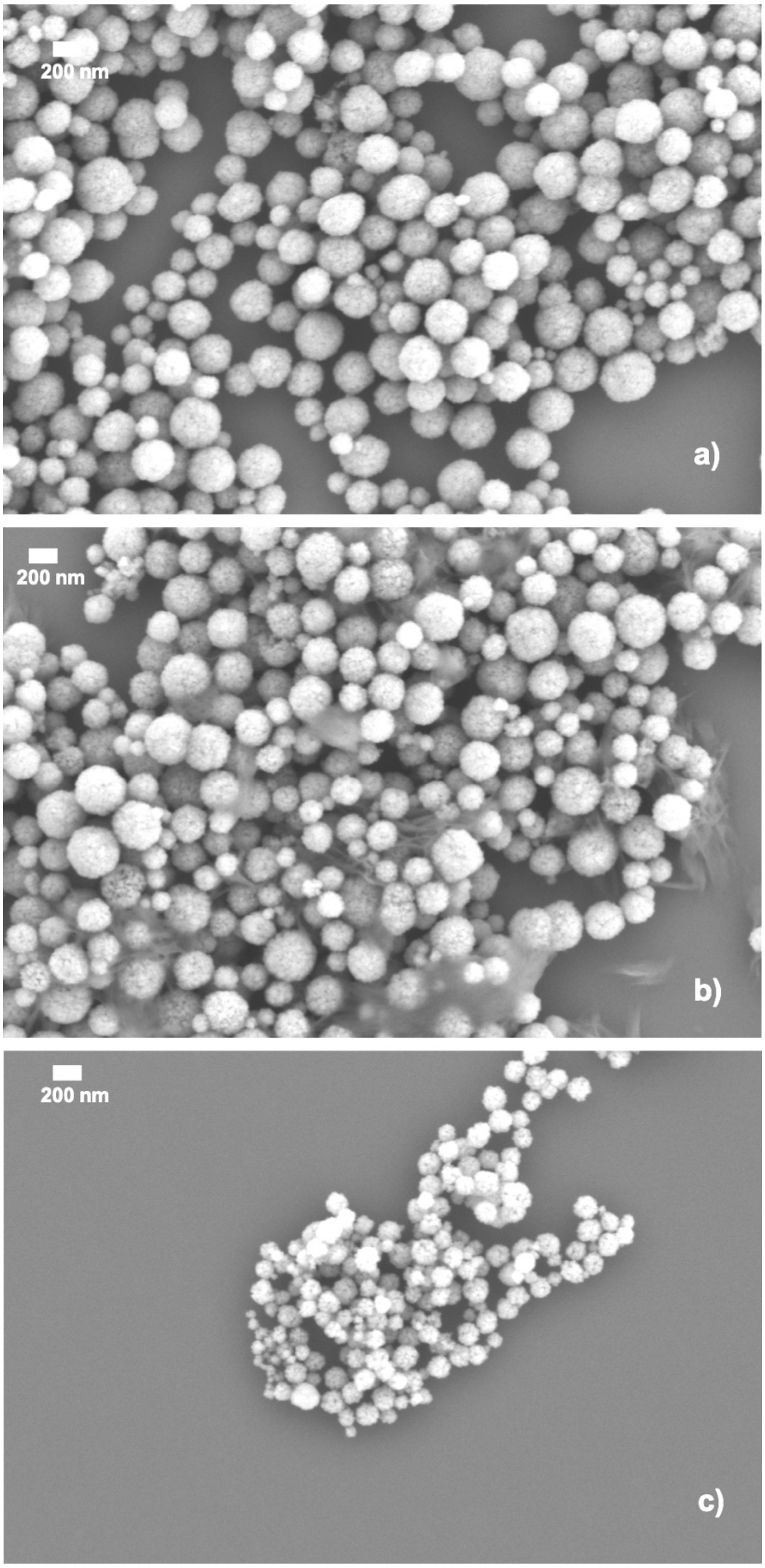

Figure S2. SEM micrographs (200 nm scale bar) of iron oxide magnetic nanoparticles prepared by solvothermal method using (**a**) no surfactant (**b**) SDS (sMNPS) (**c**) PEG (pMNPS).

2. Effect of Different Concentration of MNPS in Silica Coating

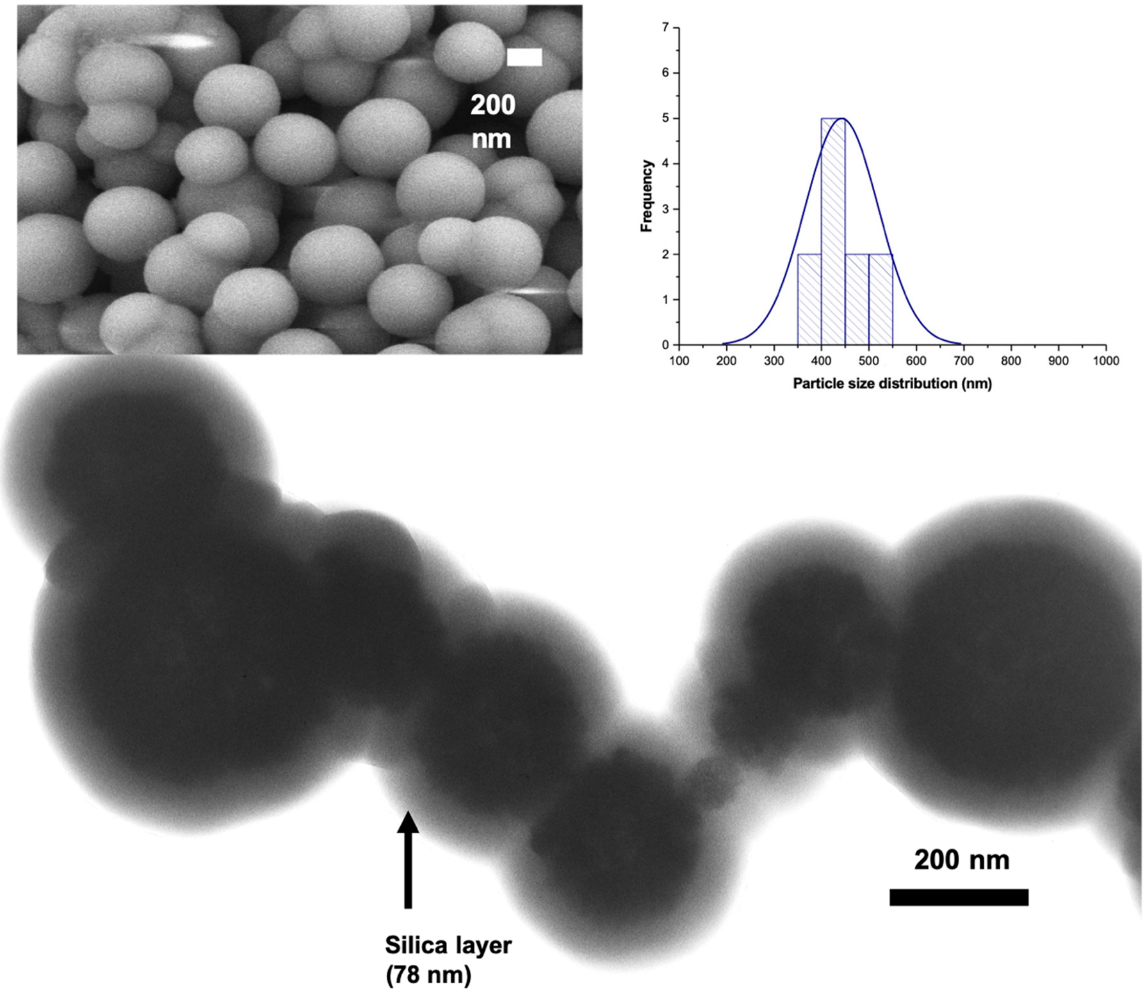

Figure S3. TEM & SEM (inset) images of sample O (0.5 mg/mL of MNPS), with particle size distribution, showing the silica layer of 78 nm encapsulating the magnetic core.

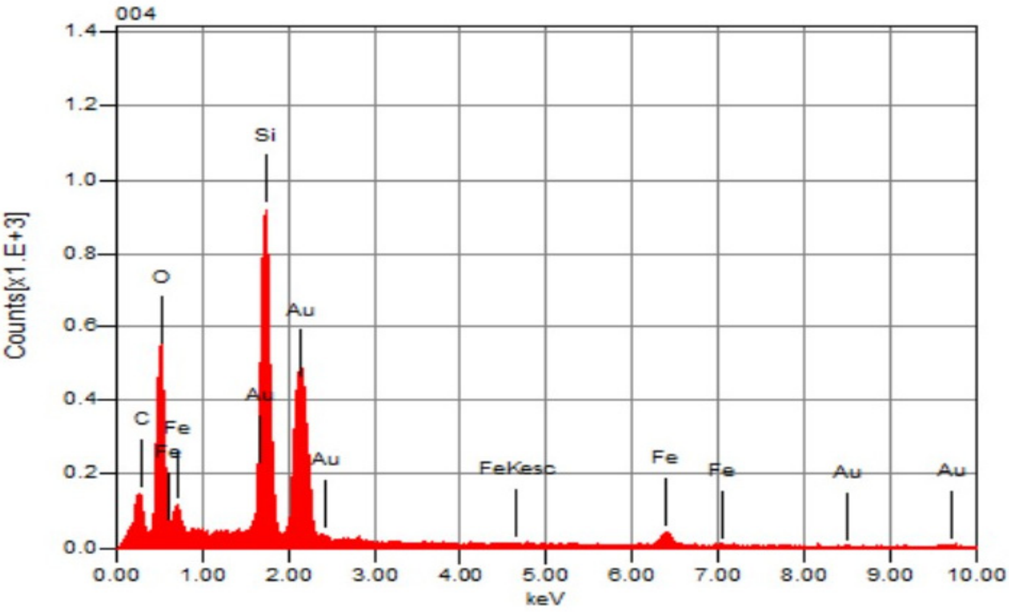

Figure S4. EDX graph confirming the presence of silica layer on the surface of magnetic nanoparticles (sample O).

3. Effect of Various TEOS Concentration on Silica Coating

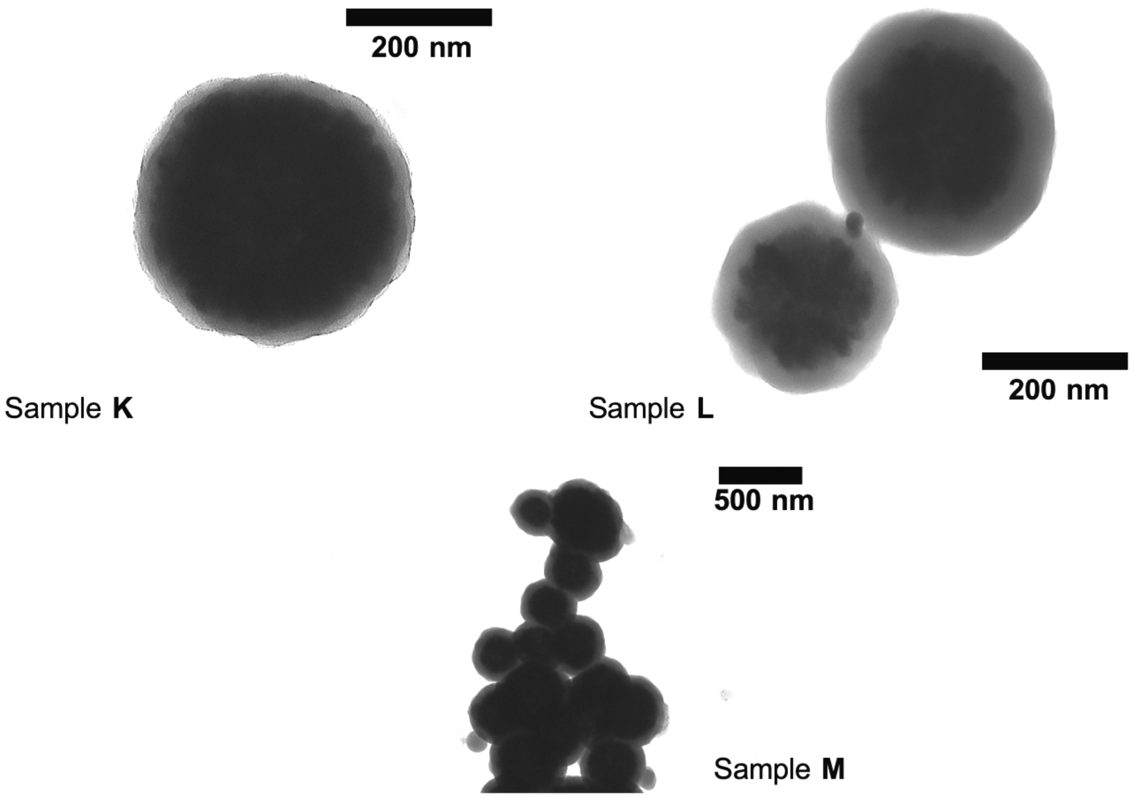

Figure S5. TEM micrographs of Sc-MNPS showing an increase in the thickness of the silica layer with respect to change in concentration of TEOS, sample K= 0.1 M, sample L= 0.25 M, sample M= 0.5 M.

4. PDI of the Sc-MNPs

Table S1. Data exhibiting polydispersity index (PDI) of the silica coated magnetic nanoparticles obtained by dynamic light scattering.

Sample Name	PDI	Silica Thickness
**A**	0.104 ± 0.033	No coating
**B**	0.116 ± 0.006	Non-uniform coating
**C**	0.087 ± 0.027	Non-uniform coating
**D**	0.099 ± 0.021	Non-uniform coating
**E**	0.095 ± 0.014	Non-uniform coating
**F**	0.152 ± 0.038	No coating
**G**	0.190 ± 0.048	Non-uniform coating
**H**	0.271 ± 0.005	30
**I**	0.111 ± 0.068	40
**J**	0.215 ± 0.015	No coating
**K**	0.230 ± 0.052	25
**L**	0.190 ± 0.116	40
**M**	0.164 ± 0.113	53
**N**	0.295 ± 0.020	Non-uniform coating
**O**	0.185 ± 0.034	18
**P**	0.245 ± 0.75	122

5. **Magnetic Hysteresis Curves**

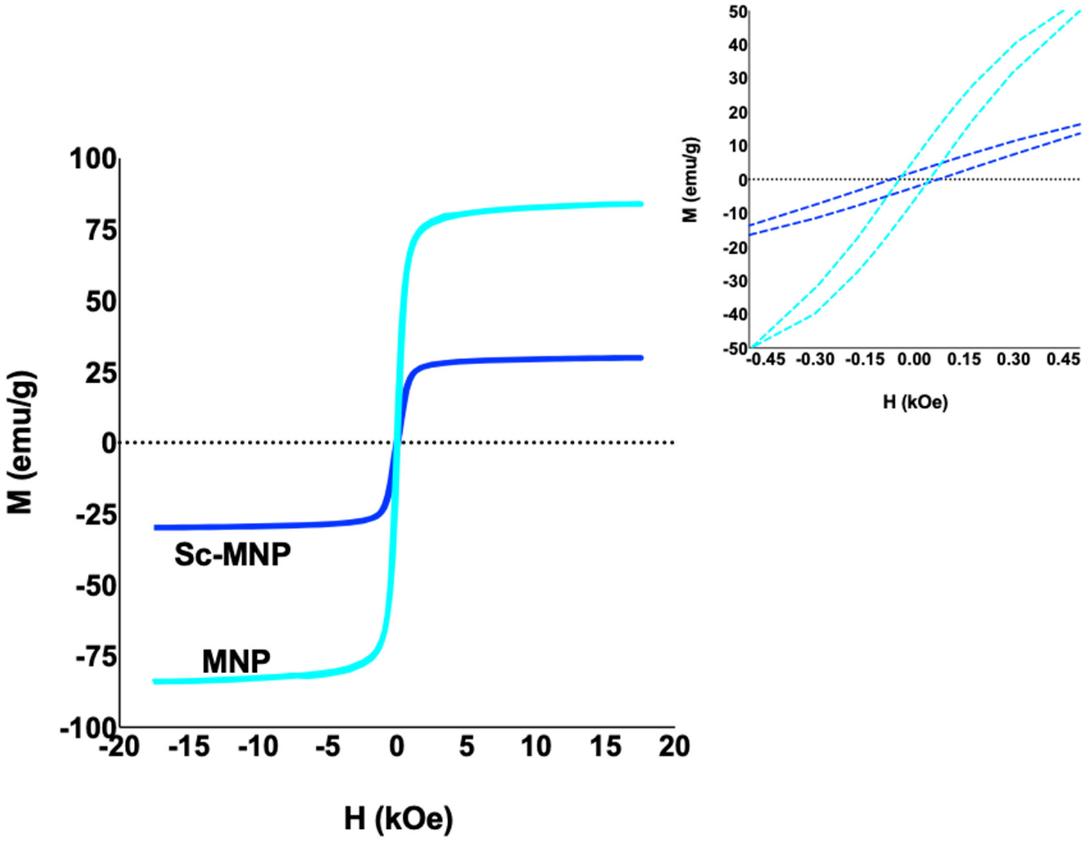

Figure S6. Magnetization hysteresis loops of MNPs (cyan) and Sc-MNP (sample P, blue) at 300 K with magnified loops inserted (H, magnetic field; M, magnetization).

6. Comparative Studies

Table S2. Comparison of material properties with those from previously reported studies.

Methodology	Solvent	Temperature (°C)	Reaction Time (h)	Surfactants Used	Z-Average (nm)	PDI	Crystal Structure
Solvothermal [1]	Ethylene glycol (25 mL)	180	6	PEG/SDS	326	-	Fe_3O_4
				PEG	343	-	
				SDS	340	-	
				No surfactant	343	-	
Solvothermal [2]	Etylene glycol (24 mL)	180	72	PEG/SDS	80	-	Fe_3O_4
				PEG	200	-	
				SDS	150	-	
				No surfactant	-	-	
Solvothermal (current work)	Ethylene glycol (150 mL)	200	24	PEG/SDS	257	0.18	γ-Fe_2O_3
				PEG	229	0.35	
				SDS	293	0.17	
				No surfactant	329	0.19	
Coprecipitation [3]	Water	60	1.5	PEG	8.6	0.17	Fe_3O_4

References

1. Huang, Y.; Zhang, L.; Huan, W.; Liang, X.; Liu, X.; Yang, Y., A study on synthesis and properties of Fe_3O_4 nanoparticles by solvothermal method. *Glass Phys. Chem*. **2010**, *36*, 325.
2. Yan, A.; Liu, X.; Qiu, G.; Wu, H.; Yi, R.; Zhang, N.; Xu, J., Solvothermal synthesis and characterization of size-controlled Fe_3O_4 nanoparticles. *J. Alloys Compd*, **2008,** *458*, 487.
3. Antarnusa, G.; Suharyadi, E., A synthesis of polyethylene glycol (peg)-coated magnetite Fe_3O_4 nanoparticles and their characteristics for enhancement of biosensor. *Mater. Res. Express*. **2020**, *7*,056103.
